# Prevalence and Correlates of Depression and Suicidal Ideation Across Stages 0–4 of Cardiovascular‐Kidney‐Metabolic Syndrome

**DOI:** 10.1002/brb3.70989

**Published:** 2025-11-11

**Authors:** Peilu Yu, Meiling Yu, Peilin Zhao, Guohui Jiang

**Affiliations:** ^1^ Department of Neurology Affiliated Hospital of North Sichuan Medical College Nanchong China; ^2^ Institute of Neurological Diseases North Sichuan Medical College Nanchong China

**Keywords:** cardiovascular‐kidney‐metabolic (CKM) syndrome, depression, mental health

## Abstract

**Background:**

The American Heart Association (AHA) recently introduced the cardiovascular‐kidney‐metabolic (CKM) syndrome framework to improve cardiovascular health management. However, evidence on the prevalence of depression and suicidal ideation (SI) across distinct CKM stages is limited.

**Methods:**

This cross‐sectional study analyzed data from 10,519 US adults participating in the National Health and Nutrition Examination Survey (NHANES) 2007 to 2018. Depression and SI were assessed using the nine‐item Patient Health Questionnaire (PHQ‐9). CKM syndrome stages were classified as: Stage 0 (absence of risk factors); Stage 1 (excess/dysfunctional adiposity); Stage 2 (metabolic risk factors or moderate‐to‐high‐risk CKD); Stage 3 (subclinical cardiovascular disease [CVD] or its risk equivalents); and Stage 4 (clinical CVD). Weighted statistical analyses were conducted to account for the complex sampling design of NHANES and obtain nationally representative estimates.

**Results:**

The prevalence of depression increased progressively with advancing CKM syndrome stages: 10.9% (Stage 0), 13.9% (Stage 1), 19.0% (Stages 2 and 3 combined), and 30.0% (Stage 4) (*p* for trend < 0.001). In contrast, the overall prevalence of SI remained relatively stable across stages (2.9%–4.9%; *p* for trend = 0.25). However, SI was significantly elevated specifically among females (8.0%) and Hispanic individuals (10.3%) in Stage 4. Stage‐specific correlates of depression varied, encompassing socioeconomic and lifestyle factors in early stages and incorporating additional complex biomedical factors in advanced stages. Depression was consistently and strongly associated with SI across all stages. Models combining stage‐specific correlates demonstrated moderate to good discrimination (area under the curve [AUC]: 0.650–0.748 for depression; 0.734–0.869 for SI).

**Conclusions:**

Advancing stages of CKM syndrome are associated with a significantly increased prevalence of depression, highlighting the critical need for integrating mental healthcare into CKM management. The identification of stage‐specific correlates provides valuable insights for developing targeted screening and intervention strategies.

## Graphical abstract

Depression prevalence rises with CKM stage (10.9%–30.0%), with suicidal ideation spiking in Stage 4 (females 8.0%, Hispanics 10.3%); correlates shift from socioeconomic/lifestyle to biomedical, necessitating mental health screening in CKM care.

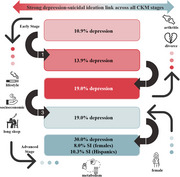

## 1 Introduction

The American Heart Association (AHA) recently introduced the concept of cardiovascular‐kidney‐metabolic (CKM) syndrome, defined as a systemic disorder characterized by complex interactions among metabolic risk factors, chronic kidney disease (CKD), and the cardiovascular system (Ndumele, Rangaswami, et al. 2023). The pathophysiological progression of CKM syndrome is categorized into five distinct stages: Stage 0 (absence of risk factors); Stage 1 (excess/dysfunctional adiposity); Stage 2 (metabolic risk factors or moderate‐to‐high‐risk CKD); Stage 3 (subclinical cardiovascular disease [CVD] or its risk equivalents, including very high‐risk CKD or high predicted 10‐year CVD risk); and Stage 4 (clinical CVD) (Ndumele, Rangaswami, et al. 2023). Nationwide epidemiological studies from the United States, the United Kingdom, and South Korea report that 80%–90% of adults meet criteria for CKM syndrome (Stage 1 or higher), with over 10% classified as Stage 3 or 4 (X. Huang, Liang, et al. 2024; Yim et al. 2025; Aggarwal et al. 2024). As a systemic disorder affecting multiple organ systems, more advanced stages of CKM syndrome are strongly associated with an increased risk of all‐cause mortality, especially Stages 3 and 4 (N. Li, Li, et al. 2024). Given the significant adverse health outcomes and socioeconomic burden associated with advanced CKM syndrome, there is an urgent need for optimized strategies aimed at preventing disease progression and implementing stage‐specific management.

Considerable attention has been focused on managing traditional cardiovascular health risk factors outlined in Life's Essential 8, including diet, physical activity, nicotine exposure, sleep health, body weight, blood lipids, blood glucose, and blood pressure (Lloyd‐Jones et al. 2022). These well‐established modifiable factors are associated with the prevalence and severity of CKM syndrome (M.‐Y. Tan, Zhang, et al. 2025). Conversely, the mental health aspects of CKM syndrome have received relatively little attention. We frame our investigation within the biopsychosocial model, which posits that depression arises from complex interactions between biological vulnerabilities (e.g., inflammatory processes, hypothalamic–pituitary–adrenal [HPA] axis dysregulation), psychological factors (e.g., coping styles, cognitive patterns), and social determinants (e.g., socioeconomic status, social support). This model is particularly relevant for understanding mental health in CKM syndrome, where progressive physiological dysregulation may interact with psychological distress and social challenges to increase depression risk. Depression, a common mental disorder, ranks as the second leading cause of disability worldwide, accounting for 56.3 million years lived with disability (YLDs) and 6.2% of global YLDs in 2021 (Ferrari et al. 2024). Accumulating evidence from cohort studies demonstrates that depression increases the risk of developing obesity (T. Liu et al. 2023), hypertension (Rosas et al. 2025), diabetes mellitus (Hu et al. 2025), metabolic syndrome (Ferriani et al. 2023), CKD (M. Liu et al. 2022), and CVD (Y. Zhang, Li, et al. 2022). However, these previous studies primarily focused on individual components of CKM syndrome and did not assess depression within the context of the integrated multi‐disease framework of CKM syndrome. Given that depression is a risk factor for the individual components of CKM syndrome, we hypothesized that its prevalence of depression would increase with advancing CKM stage. Therefore, this study aimed to investigate the prevalence of depression across CKM Stages 0–4 and identify stage‐specific predictive factors to inform targeted screening and interventions. In addition, given the high prevalence of suicidal ideation among individuals with depression (Cai et al. 2021), we also evaluated suicidal ideation as a secondary mental health outcome.

In summary, leveraging nationally representative data from the National Health and Nutrition Examination Survey (NHANES), this study aimed to: (1) estimate the prevalence of depression and suicidal ideation across Stages 0–4 of CKM syndrome in US adults; (2) assess the association between CKM stage and depression; (3) identify factors independently associated with depression and suicidal ideation within each CKM stage; and (4) develop and evaluate stage‐specific predictive models for these mental health outcomes.

## 2 Materials and Methods

**FIGURE 1:**
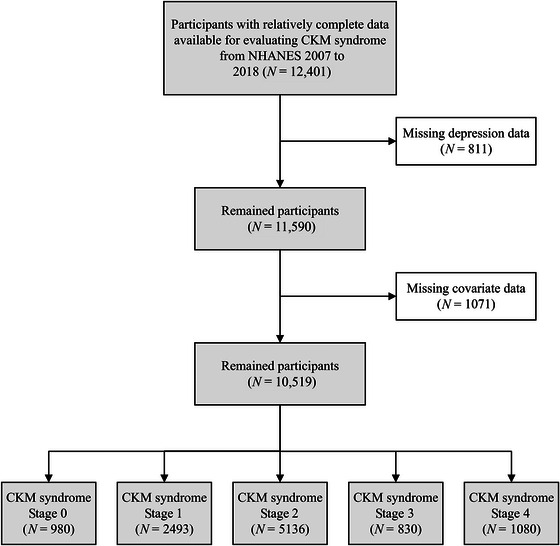
Flowchart of participant selection process.

NHANES is dedicated to research on health and disease to advance public health policies and improve health program services. The National Center for Health Statistics Ethics Review Board approved the survey procedures, and all participants provided informed consent. Following the CKM syndrome staging assessment methodology previously adapted for NHANES data analysis (Aggarwal et al. 2024), we included adults aged ≥ 20 years from the NHANES fasting subsample. Considering the data availability on depression, suicidal ideation, and relevant factors, participants from NHANES cycles between 2007 and 2018 were initially included (*N* = 12,401). Participants with missing data on depressive symptoms (*N* = 811) or relevant covariates (*N* = 1071) were excluded, resulting in a final analytical sample of 10,519 participants (Figure 1). The detailed definitions and assessment methods of CKM syndrome Stages 0−4 are provided in Table S1.

NHANES collected comprehensive data, including socioeconomic characteristics, lifestyle habits, health status, physical examination findings, and laboratory test results. Data were extracted for the following purposes: (1) evaluation of CKM syndrome staging, (2) assessment of depression and suicidal ideation, and (3) identification of their associated factors. The sociodemographic variables assessed included: age; sex (male, female); race/ethnicity (Hispanic, non‐Hispanic White, non‐Hispanic Black, other); education level (less than high school, high school graduate or equivalent, some college or higher); marital status (married/living with partner, widowed/divorced/separated, never married); family income‐to‐poverty ratio (PIR: < 1.3, 1.3–3.5, > 3.5); home ownership; and health insurance coverage. Lifestyle factors assessed were: current smoking status; physical inactivity; and sleep duration categorized as short (< 7 h), recommended (7–9 h), or long (> 9 h). Health status indicators included: estimated glomerular filtration rate (eGFR); hypertension; diabetes mellitus; metabolic syndrome; CVD; cancer; arthritis; depressive symptoms; and current use of antihypertensive medications, statins, or antidepressants. Physical examinations included body mass index, waist circumference, and systolic and diastolic blood pressure. Laboratory tests included fasting blood glucose, glycated hemoglobin, serum creatinine, triglycerides, total cholesterol, high‐density lipoprotein cholesterol (HDL‐C), and urinary albumin‐to‐creatinine ratio. Detailed definitions of the indicators for lifestyle habits and health status are listed in Table S2.

Depressive symptoms over the preceding 2 weeks were assessed using the nine‐item Patient Health Questionnaire (PHQ‐9). A score of ≥ 10 on the PHQ‐9, which has a sensitivity and specificity of 88% for identifying major depression (Kroenke et al. 2001), was used to define depression. Suicidal ideation was defined as an affirmative response (“several days,” “more than half the days,” or “nearly every day”) to item 9 of the PHQ‐9: “Over the last 2 weeks, how often have you been bothered by thoughts that you would be better off dead or of hurting yourself in some way?” (H. Huang, Fu, et al. 2024; Lv et al. 2024).

To account for the complex, multistage probability sampling design of NHANES and to generate nationally representative estimates, appropriate fasting subsample weights, stratification variables, and primary sampling units were incorporated into all analyses as recommended (Johnson et al. 2013). Descriptive statistics (weighted proportions for categorical variables, weighted medians and interquartile ranges [IQRs] for continuous variables) were calculated overall and stratified by CKM stage. Differences in participant characteristics across CKM stages were assessed using Rao–Scott chi‐square tests for categorical variables and appropriate nonparametric tests accounting for the survey design (e.g., Kruskal–Wallis for > 2 groups) for continuous variables. Within each CKM stage, characteristics were compared between participants with versus without depression and with versus without suicidal ideation using Rao–Scott chi–square tests (categorical) and Wilcoxon rank‐sum tests (continuous), appropriately weighted for the complex survey design. Binary multivariable logistic regression models, adjusted for socioeconomic characteristics (age, sex, race/ethnicity, education, marital status, PIR), were used to examine the association between CKM stage (modeled ordinally) and the odds of depression and suicidal ideation. Lifestyle and health status variables were not included in these models due to their potential role as mediators or colliders on the causal pathway between CKM stage and mental health outcomes. Within each CKM stage, separate multivariable logistic regression models were fitted to identify factors independently associated with depression and suicidal ideation. Variables showing an association with the outcome at *p* < 0.2 in univariate analyses were considered for inclusion in the initial multivariable model for that stage. Backward selection based on the likelihood ratio test (retention criterion *p* < 0.05) or clinical relevance was used to derive the final parsimonious model for each outcome within each stage. The predictive performance of the final set of independent correlates for each outcome within each stage was evaluated by calculating the area under the receiver operating characteristic curve (AUC). All analyses accounted for the NHANES complex survey design and were performed using R software (version 4.4.2) with the “survey” package. Statistical significance was defined as a two‐sided *p* value < 0.05.

## 3 Results

The final analytical sample comprised 10,519 participants, distributed across CKM stages as follows: Stage 0, 980 (11.2%); Stage 1, 2493 (27.1%); Stage 2, 5136 (48.8%); Stage 3, 830 (4.9%); and Stage 4, 1080 (8.0%). Weighted participant characteristics stratified by CKM stage are presented in Table 1. Participants in more advanced CKM stages were significantly older, had a higher proportion of males and non‐Hispanic White individuals, and exhibited less favorable social determinants of health, including lower educational attainment, lower household income (lower PIR), and higher rates of widowed/divorced/separated status. They also reported poorer lifestyle habits, such as higher rates of current smoking and physical inactivity, and a higher prevalence of insufficient sleep. Furthermore, the prevalence of comorbidities, including cancer and arthritis, was significantly higher in advanced stages.

**TABLE 1:** Characteristics of participants stratified by CKM syndrome stages.

Characteristic	Stage 0, *N* = 980 (11.2%)	Stage 1, *N* = 2493 (27.1%)	Stage 2, *N* = 5136 (48.8%)	Stage 3, *N* = 830 (4.9%)	Stage 4, *N* = 1080 (8.0%)	*p* value
Age, years	31.0 (25.0–43.0)	38.0 (28.0–50.0)	49.0 (36.0–59.0)	77.0 (72.0–80.0)	66.0 (57.0–75.0)	< 0.001
Sex, %						< 0.001
Male	369 (36.8)	1196 (49.7)	2603 (52.0)	490 (52.6)	627 (55.7)	
Female	611 (63.2)	1297 (50.3)	2533 (48.0)	340 (47.4)	453 (44.3)	
Race, %						< 0.001
Hispanic	202 (11.5)	687 (15.9)	1464 (14.7)	152 (9.6)	192 (8.3)	
Non‐Hispanic White	489 (73.5)	1025 (67.5)	2153 (69.2)	459 (75.1)	601 (74.9)	
Non‐Hispanic Black	158 (9.0)	510 (11.3)	995 (10.3)	169 (11.0)	235 (11.7)	
Other	131 (6.0)	271 (5.3)	524 (5.8)	50 (4.2)	52 (5.0)	
Education level, %						< 0.001
Less than high school	129 (8.9)	466 (12.0)	1232 (15.7)	256 (24.0)	333 (21.9)	
High school	195 (19.1)	499 (19.8)	1218 (23.9)	231 (30.7)	275 (27.2)	
More than high school	656 (72.0)	1528 (68.1)	2686 (60.3)	343 (45.4)	472 (50.9)	
Marital status, %						< 0.001
Married/living with partner	515 (57.6)	1528 (64.9)	3236 (66.3)	481 (59.0)	631 (64.5)	
Widowed/divorced/separated	101 (7.7)	353 (12.5)	1097 (18.2)	319 (37.7)	378 (30.3)	
Never married	364 (34.7)	612 (22.6)	803 (15.6)	30 (3.3)	71 (5.1)	
PIR, %						< 0.001
> 3.5	349 (48.7)	839 (44.5)	1607 (42.8)	187 (29.1)	241 (30.9)	
< 1.3	289 (20.2)	733 (20.2)	1610 (21.1)	256 (23.6)	382 (25.9)	
1.3−3.5	342 (31.1)	921 (35.3)	1919 (36.1)	387 (47.3)	457 (43.2)	
Owning house, %	502 (58.7)	1469 (65.5)	3229 (69.0)	632 (81.5)	739 (75.7)	< 0.001
Medical insurance coverage, %	718 (80.6)	1847 (80.7)	3929 (82.3)	797 (96.9)	980 (91.5)	< 0.001
Current smoking, %	202 (19.6)	480 (17.8)	1098 (20.3)	115 (11.4)	246 (23.0)	< 0.001
Physical inactivity, %	241 (20.8)	728 (26.6)	1953 (34.9)	486 (56.6)	546 (46.3)	
Sleep duration, %						< 0.001
7−9 h	668 (71.5)	1584 (68.7)	3036 (62.7)	528 (66.6)	605 (61.4)	
< 7 h	266 (24.1)	811 (27.9)	1869 (32.9)	231 (25.3)	390 (31.8)	
> 9 h	46 (4.5)	98 (3.4)	231 (4.4)	71 (8.1)	85 (6.9)	
Body mass index, kg/m^2^	22.0 (20.4–23.4)	27.4 (25.1–30.6)	29.7 (26.2–34.2)	28.2 (25.3–32.2)	29.3 (25.6–34.1)	< 0.001
GFR, mL/min/1.73 m^2^	107.8 (94.5–120.7)	104.2 (90.9–116.5)	100.0 (85.7–111.5)	67.8 (54.1–83.5)	81.9 (64.7–96.2)	< 0.001
Hypertension, %	0 (0.0)	0 (0.0)	2821 (54.2)	724 (87.3)	835 (73.8)	< 0.001
Diabetes mellitus, %	0 (0.0)	0 (0.0)	1071 (16.2)	416 (46.3)	444 (35.8)	< 0.001
Metabolic syndrome, %	0 (0.0)	0 (0.0)	3076 (60.0)	546 (66.0)	691 (62.6)	< 0.001
Cancer, %	40 (5.8)	109 (5.3)	379 (8.5)	202 (29.9)	236 (23.1)	< 0.001
Arthritis, %	77 (9.3)	354 (15.2)	1439 (28.4)	404 (49.8)	602 (54.6)	< 0.001
Depression, %	97 (10.9)	282 (13.9)	892 (19.0)	147 (19.0)	308 (30.0)	< 0.001
Antidepressant use, %	56 (7.8)	173 (9.9)	574 (14.3)	105 (14.9)	209 (23.1)	< 0.001
PHQ‐9 score	1.0 (0.0–3.0)	1.0 (0.0–4.0)	2.0 (0.0–4.0)	2.0 (0.0–4.0)	3.0 (0.0–6.0)	< 0.001
Suicidal ideation, %	30 (2.9)	64 (3.0)	186 (3.1)	27 (3.9)	63 (4.9)	0.25

Abbreviations: CKM, cardiovascular‐kidney‐metabolic; GFR, glomerular filtration rate; PHQ‐9, nine‐item Patient Health Questionnaire; PIR, family income‐to‐poverty ratio.

As shown in Table 1, the prevalence of depression demonstrated a marked graded increase with advancing CKM stage: 10.9% (Stage 0), 13.9% (Stage 1), 19.0% (Stages 2 and 3 combined), and 30.0% (Stage 4) (*p* for trend < 0.001). Consistent with this trend, rates of antidepressant use and mean PHQ‐9 scores also increased significantly across stages (*p* for trend < 0.001 for both, Table 1). In contrast to depression, the overall prevalence of suicidal ideation did not show a significant trend across CKM stages (range 2.9%–4.9%, *p* for trend = 0.25, Table 1). Table 2 presents the prevalence of depression and suicidal ideation stratified by sex and race/ethnicity. The progressive increase in depression prevalence with advancing CKM stage was observed consistently across all sex and racial/ethnic subgroups (all *p* for trend < 0.001, Table 2). In contrast, while the overall prevalence of suicidal ideation showed no significant trend, subgroup analyses revealed that among individuals with Stage 4 CKM syndrome, females had a significantly higher prevalence of suicidal ideation than males (8.0% vs. 2.5%, *p* < 0.01), and Hispanic individuals had a significantly higher prevalence than other racial/ethnic groups (10.3% vs. 5.3% in non‐Hispanic White, 1.3% in non‐Hispanic Black, and 0.0% in other, *p* < 0.001). After adjusting for socioeconomic characteristics, the likelihood of participants developing depression remained positively correlated with CKM syndrome stages, showing a significant trend (*p* for trend < 0.001, Table S3). No significant differences were observed in antidepressant usage rates, disease severity (PHQ‐9 scores), or prevalence of suicidal ideation in depression populations in different stages of CKM syndrome (Table S4).

**TABLE 2:** The prevalence of depression and suicidal ideation across CKM syndrome stages.

Subgroup	Stage 0	Stage 1	Stage 2	Stage 3	Stage 4	*p* value
Depression						
Sex						
Male	22/369 (5.9%)	77/1196 (7.0%)	317/2603 (13.3%)	67/490 (12.9%)	122/627 (19.8%)	< 0.001
Female	75/611 (13.8%)	205/1297 (20.7%)	575/2533 (25.3%)	80/340 (25.8%)	186/453 (42.7%)	< 0.001
Race						
Hispanic	21/202 (8.4%)	62/687 (7.7%)	198/1464 (12.9%)	24/152 (16.8%)	53/192 (27.9%)	< 0.001
Non‐Hispanic White	54/489 (11.8%)	169/1025 (16.5%)	494/2153 (21.1%)	87/459 (19.6%)	184/601 (30.0%)	< 0.001
Non‐Hispanic Black	15/158 (8.8%)	39/510 (9.5%)	139/995 (14.6%)	30/169 (19.7%)	58/235 (29.0%)	< 0.001
Other	7/131 (7.5%)	12/271 (8.9%)	61/524 (17.7%)	6/50 (12.2%)	13/52 (35.3%)	0.004
Suicidal ideation						
Sex						
Male	13/369 (2.9%)	33/1196 (2.8%)	97/2603 (3.3%)	18/490 (4.7%)	23/627 (2.5%)	0.70
Female	17/611 (2.9%)	31/1297 (3.1%)	89/2533 (3.0%)	9/340 (2.9%)	40/453 (8.0%)	0.004
Race						
Hispanic	7/202 (3.6%)	19/687 (2.5%)	67/1464 (4.8%)	5/152 (2.6%)	24/192 (10.3%)	< 0.001
Non‐Hispanic White	15/489 (2.9%)	28/1025 (3.0%)	79/2153 (2.7%)	14/459 (4.0%)	36/601 (5.3%)	0.22
Non‐Hispanic Black	7/158 (3.0%)	11/510 (2.6%)	26/995 (2.7%)	5/169 (3.2%)	3/235 (1.3%)	0.86
Other	1/131 (0.3%)	6/271 (4.0%)	14/524 (4.7%)	3/50 (5.9%)	0/52 (0.0%)	0.34

Abbreviation: CKM, cardiovascular‐kidney‐metabolic.

**FIGURE 2:**
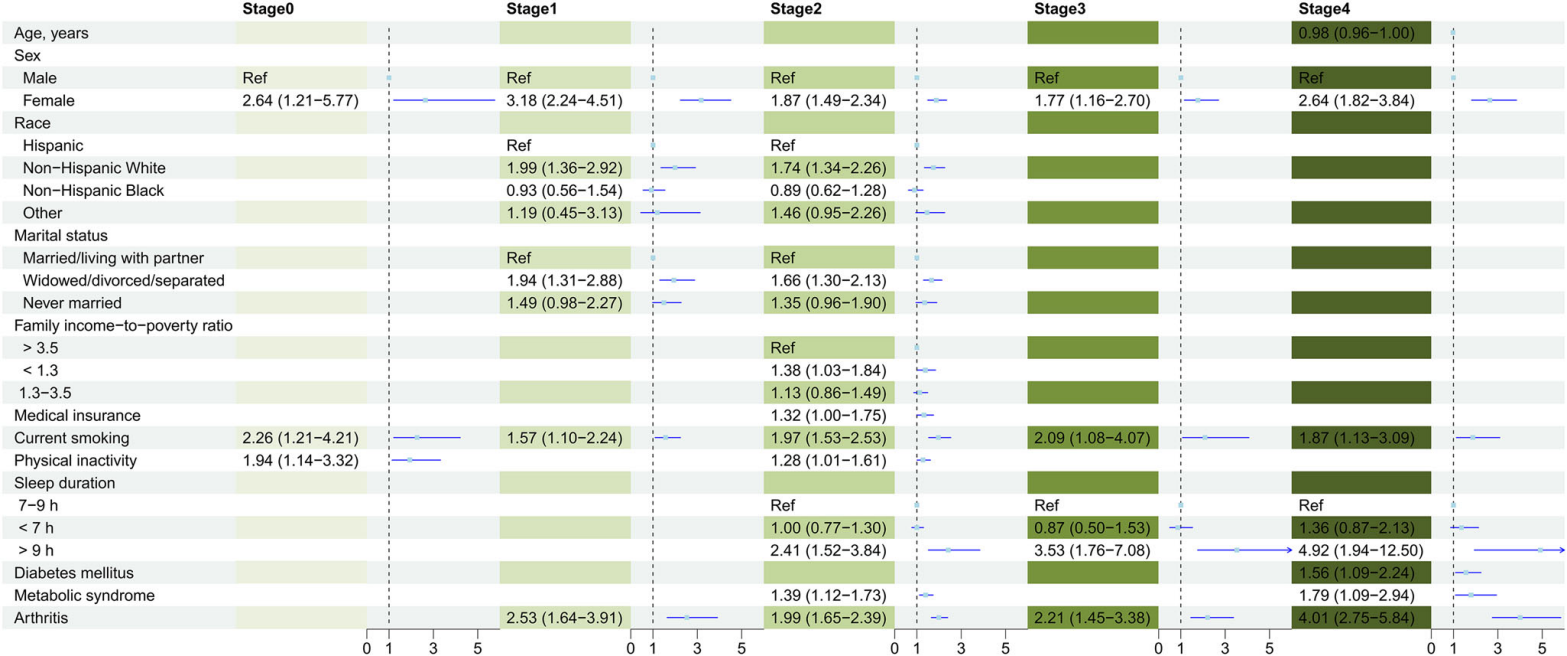
Forest plot showing independent correlates of depression across cardiovascular‐kidney‐metabolic syndrome stages 0–4.

**FIGURE 3:**
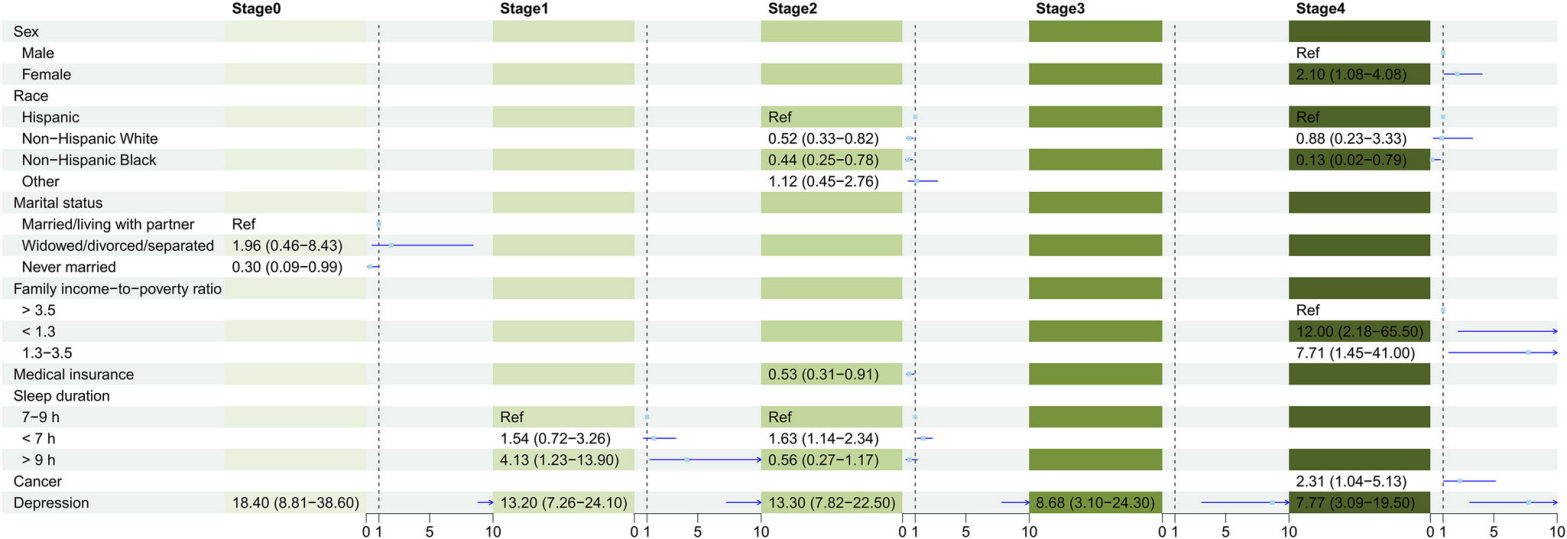
Forest plot showing independent correlates of suicidal ideation across cardiovascular‐kidney‐metabolic syndrome stages 0–4.

**FIGURE 4:**
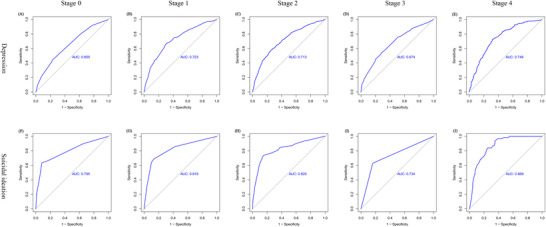
The predictive efficacy of a combination of independent correlates for depression and suicidal ideation across different CKM syndrome stages.

Figures 2 and 3 display the factors independently associated with depression and suicidal ideation, respectively, within each CKM stage, based on multivariable logistic regression analyses. Female sex and current smoking status were consistently and independently associated with depression across all CKM stages. Depression was consistently and strongly associated with suicidal ideation in every stage. Beyond these common factors, numerous other variables demonstrated stage‐specific associations with both outcomes. Significant independent correlates for depression included: physical inactivity (Stage 0); non‐Hispanic White race, widowed/divorced/separated status, and arthritis (Stage 1); non‐Hispanic White race, widowed/divorced/separated status, lower PIR (income), health insurance coverage, physical inactivity, long sleep duration (> 9 h), metabolic syndrome, and arthritis (Stage 2); long sleep duration (> 9 h) and arthritis (Stage 3); and younger age (< 65 years, interpretation based on result), long sleep duration (> 9 h), diabetes mellitus, metabolic syndrome, and arthritis (Stage 4) (see Figure 2 for odds ratios and confidence intervals). Significant independent correlates for suicidal ideation included: widowed/divorced/separated status (Stage 0); long sleep duration (> 9 h) (Stage 1); Hispanic race/ethnicity, lack of health insurance, and short sleep duration (< 7 h) (Stage 2); and female sex, Hispanic race/ethnicity, lower PIR, and cancer (Stage 4) (Stage 3 showed no significant correlates beyond depression) (see Figure 3 for odds ratios and confidence intervals). The predictive performance of the combined sets of stage‐specific independent correlates is shown in Figure 4. For depression, the AUC ranged from 0.650 (Stage 1) to 0.748 (Stage 5), indicating moderate discrimination. For suicidal ideation, AUC values were higher, ranging from 0.734 (Stage 4) to 0.869 (Stage 5), indicating good to excellent discrimination across most stages.

## 4 Discussion

This study presents the first comprehensive assessment of depression and suicidal ideation across the entire spectrum of CKM syndrome Stages (0–4) in a nationally representative US adult population. Key findings include: (1) a marked graded increase in depression prevalence with advancing CKM stage, nearly tripling from Stage 0 (10.9%) to Stage 4 (30.0%); (2) a stable overall prevalence of suicidal ideation across stages (2.9%–4.9%), but significantly elevated rates specifically among females (8.0%) and Hispanic individuals (10.3%) with Stage 4 disease; and (3) the identification of distinct, stage‐specific profiles of factors independently associated with depression and suicidal ideation, evolving from predominantly socioeconomic and lifestyle factors in early stages to include complex biomedical comorbidities in advanced stages. By utilizing the novel integrated CKM staging framework, this work significantly extends previous research focused on individual disease components and offers novel insights into mental health burdens within the context of multisystem chronic disease progression.

Due to the interactions between metabolic risk factors, CKD, and the cardiovascular system, the CKM syndrome framework was proposed to address the interconnected nature of these conditions. Overlapping pathophysiological processes, including inflammation, oxidative stress, insulin resistance, and vascular dysfunction, drive the progression of CKM syndrome, ultimately leading to CVD and kidney failure (Ndumele, Neeland, et al. 2023). The AHA presidential advisory recommends applying Life's Essential 8 framework to promote cardiovascular health and systematically screening for social determinants of health in patients with CKM syndrome (Ndumele, Rangaswami, et al. 2023). However, few studies have examined the link between CKM syndrome and depression. A large prospective cohort study of 344,956 UK Biobank participants found that poor CKM health is independently associated with an increased risk of depression over a median follow‐up of 12.8 years (Huang et al. 2024). The association between depression and CKM syndrome is likely bidirectional, as depression increases the risk of individual components of CKM syndrome (T. Liu et al. 2023; Rosas et al. 2025; Hu et al. 2025; Ferriani et al. 2023; M. Liu et al. 2022; Y. Zhang, Li, et al. 2022). This bidirectional association results in a significant dose–response relationship between CKM syndrome stages and the prevalence of depression, although no difference in depression severity was observed across stages. Compared to previous findings on suicidal ideation and individual CKM components (Yu et al. 2025; Motsa et al. 2021; Ko et al. 2019; M.‐Z. Zhang, Tang, et al. 2022; Elamoshy et al. 2018), this study found no overall difference in suicidal ideation prevalence across CKM stages, except within specific subgroups (females and Hispanics) at Stage 4. Discrepancies in results may be attributed to heterogeneity in study populations and different assessment methods for suicidal ideation. The higher prevalence of depression in females and distinct sociocultural stressors experienced by Hispanic populations could account for their heightened vulnerability to suicidal ideation (Danzo et al. 2024).

The precise mechanisms underpinning the bidirectional relationship between depression and CKM syndrome remain incompletely understood but likely involve intertwined pathways. Shared core pathophysiological processes of CKM syndrome, such as chronic inflammation and oxidative stress (Ndumele, Neeland, et al. 2023), are known to disrupt neurotransmitter systems (e.g., serotonin, dopamine) and impair neural circuitry regulating mood and motivation, thereby contributing to depression (Miller and Raison 2016).

Concurrently, the physiological burden and psychological distress associated with CKM syndrome can activate the HPA axis, further exacerbating depressive symptoms (Tang et al. 2018). Conversely, depression is associated with adverse health behaviors—including poor diet, physical inactivity, smoking, and sleep disturbances (Bai and Guo 2024)—that directly impair cardiovascular, metabolic, and renal health, fueling a vicious cycle that accelerates CKM progression. Notably, depression itself may induce metabolic dysregulation, such as insulin resistance, even in the absence of overt diabetes, thereby increasing CKM risk (Z. Tan, Zhou, et al. 2025; Lee et al. 2017). Furthermore, both conditions share upstream social determinants of health (e.g., low socioeconomic status, limited education, social isolation) (Kirkbride et al. 2024; J. Li, Lei, et al. 2024). Crucially, our finding that the association between advanced CKM stage and depression persisted after adjustment for key socioeconomic factors strongly suggests that direct pathophysiological interactions beyond shared social risk factors contribute significantly to this relationship.

The steep rise in depression prevalence with advancing CKM stage underscores the critical need for systematic screening and integrated management of mental health in this population, aligning with principles of integrative psychosomatic medicine (Deter et al. 2018). Evidence suggests that treating comorbid depression in patients with CVD can improve clinical outcomes, including reduced hospitalizations and mortality (Carmin et al. 2024). Consequently, addressing depressive symptoms alongside traditional CKM risk factors may not only alleviate mental suffering but also potentially mitigate CKM progression. Our identification of stage‐specific correlates provides a roadmap for targeted interventions. In early CKM Stages (0–2), interventions should prioritize addressing socioeconomic stressors, promoting healthy lifestyles (increasing physical activity, optimizing sleep), and managing conditions like arthritis. In advanced stages (Stages 3 and 4), while lifestyle interventions remain important, optimizing management of core CKM components (diabetes, metabolic syndrome) and addressing pain (arthritis) are also crucial for reducing depression risk; particular attention should be paid to younger patients struggling with the impact of advanced disease. The identified correlates, particularly modifiable lifestyle and biomedical factors, offer tangible targets for mental health interventions tailored to the CKM stage. Furthermore, given the strong and consistent association between depression and suicidal ideation, screening for suicidal thoughts is essential in individuals identified as high risk for or diagnosed with depression, especially within the high‐risk subgroups identified in Stage 4 (females, Hispanics, those with low income or cancer).

Strengths of this study include its large, nationally representative sample, the application of the novel integrated CKM staging framework, standardized assessment of mental health outcomes, and rigorous survey‐weighted analyses. However, several limitations warrant consideration. First, the use of US NHANES data limits generalizability to other populations with differing racial/ethnic compositions, sociocultural contexts, or healthcare systems; external validation is needed. Second, depression was defined by PHQ‐9 score ≥ 10, not a clinician‐administered structured diagnostic interview. While the PHQ‐9 is a reliable and valid screening tool well suited for large surveys and primary care (Costantini et al. 2021), it may over‐ or under‐identify clinical depression compared to gold‐standard assessments. Third, important psychosocial determinants of mental health (e.g., specific personality traits, detailed measures of social support or isolation, chronic stress levels) were not available in NHANES (Altaweel et al. 2023; De Risio et al. 2024), limiting the comprehensiveness of our correlates and potentially the predictive accuracy of our models. Future studies incorporating these factors are warranted. Fourth, and most importantly, the cross‐sectional design precludes causal inference regarding the directionality of the observed associations between CKM stage and mental health outcomes. Prospective cohort studies are essential to elucidate whether depression accelerates CKM progression and to establish the temporal sequence of these complex relationships.

## 5 Conclusions

This study provides compelling evidence that depression prevalence increases markedly with advancing CKM syndrome stage, while suicidal ideation risk rises sharply in specific vulnerable subgroups (females, Hispanics) during Stage 4. The delineation of distinct, stage‐specific risk profiles—evolving from socioeconomic and lifestyle factors in early stages to complex biomedical comorbidities in advanced stages—offers crucial insights for developing targeted screening and preventive strategies. Furthermore, the demonstrated predictive performance (AUC 0.65–0.87) of models incorporating these stage‐specific factors supports the feasibility of implementing tailored mental health screening within CKM management protocols. These findings strongly advocate for the integration of routine depression assessment into the care of individuals with CKM syndrome. Future research should prioritize investigating whether evidence‐based mental health interventions can effectively slow CKM progression and improve overall clinical outcomes in this high‐risk population.

## Author Contributions

**Peilu Yu**: data curation, formal analysis, investigation, methodology, visualization, writing – original draft. **Meiling Yu**: methodology, visualization. **Peilin Zhao**: methodology, funding acquisition. **Guohui Jiang**: conceptualization, project administration, supervision, validation, writing – review and editing.

## Funding

Sichuan Science and Technology Program (Grant No.2023NSFSC1575).

## Conflicts of Interest

The authors declare no conflicts of interest.

## Supporting information



Supplementary Table: brb370989‐sup‐0001‐Table.doc

Supplementary Table: brb370989‐sup‐0002‐Table.doc

Supplementary Table: brb370989‐sup‐0003‐Table.doc

Supplementary Table: brb370989‐sup‐0004‐Table.doc

## Data Availability

The data underlying this study are publicly available from the National Health and Nutrition Examination Survey (NHANES), conducted by the National Center for Health Statistics (NCHS), Centers for Disease Control and Prevention (CDC). All datasets used in this analysis can be accessed and downloaded free of charge at: https://www.cdc.gov/nchs/nhanes/.
