# Supplementary material for: Prevalence and Correlates of Depression and Suicidal Ideation Across Stages 0–4 of Cardiovascular‐Kidney‐Metabolic Syndrome
Source: Brain Behav. 2025 Nov 11;15(11):e70989. doi: 10.1002/brb3.70989 (PMC12605968; doi:10.1002/brb3.70989)
Supplement: Supplementary file 1 — Supplementary Table: brb370989‐sup‐0001‐Table.doc [file BRB3-15-e70989-s003.doc]

Table S1. Definitions of CKM syndrome stages adapted to data available in NHANES

| **CKM syndrome stages** | **Definitions** |
| --- | --- |
| Stage 0: No CKM  risk factors | Participants with normal body mass index (BMI) (<23 kg/m^2^ for individuals with Asian ethnicity and <25 kg/m^2^ for other racial and ethnic groups), normal waist circumference (<80 and <90 cm for women and men with Asian race, respectively, and <88 and <102 cm for women and men in all other race and ethnicity categories, respectively) who did not meet criteria for the other stages. |
| Stage 1: Excess  or dysfunctional  adiposity | Individuals with an elevated BMI (≥23 kg/m^2^ for individuals with Asian race and ≥25 kg/m^2^ for all other race and ethnic groups), an elevated waist circumference (≥80 and ≥90 cm for women and men with Asian race, respectively, and ≥88 and ≥102 cm for women and men in other race and ethnicity categories, respectively), or prediabetes (defined as a glycated hemoglobin of 5.7% to <6.5% or a fasting blood glucose of 100 mg/dL to <124 mg/dL). |
| Stage 2: Metabolic  risk factors and CKD | Participants with metabolic risk factors or moderate-to-high-risk CKD per Kidney Disease Improving Global Outcomes (KDIGO) criteria, as recommended by the AHA. Qualifying metabolic risk factors included elevated fasting serum triglycerides (≥135 mg/dL), hypertension, diabetes, or metabolic syndrome (≥3 of the following: elevated waist circumference, low high density lipoprotein cholesterol (HDL) level [<40 mg/dL or <50 mg/dL for men or women, respectively], fasting serum triglycerides ≥150 mg/dL, elevated blood pressure [systolic blood pressure ≥130, diastolic blood pressure ≥80 mmHg, and/or use of blood pressure-lowering medications], or prediabetes). CKD stages were identified based on GFR and urinary albumin-to-creatinine ratio. |
| Stage 3: Subclinical  CVD in CKM | The presence of very-high-risk KDIGO CKD stages or a high-predicted 10-year CVD risk. 10-year cardiovascular risk was estimated with the AHA Predicting Risk of CVD EVENTs (PREVENT) equations. 3 High risk was defined as ≥20% 10-year CVD risk (based on recommended thresholds [https://professional.heart.org/en/guidelines-and-statements/prevent-calculator]). The PREVENT equations were developed and validated for adults 30-79 years of age. As such, risk was not estimated for adults <30 years. However, to minimize underestimation of CKD Stage 3, adults ≥80 years were not excluded from 10-year CVD risk. Instead, adults ≥80 years were assigned an age of 79 years when determining 10-year CVD risk to allow for conservative estimates. Further, PREVENT was developed for variables with the following ranges: total cholesterol 130-320 mg/dL, HDL 20-100 mg/dL, systolic blood pressure 90-200 mmHg, and GFR 14-140 mL/min/1.73m². To approximate PREVENT risk strata, values for these variables above or below these bounds were set to the upper or lower bounds of allowable values respectively (for example, total cholesterol of 330 mg/dL was set as 320 mg/dL). Cardiac biomarkers and cardiovascular imaging were not available to identify subclinical CVD. |
| Stage 4: Clinical  CVD in CKM | Self-reported established cardiovascular disease (coronary heart disease, angina, heart attack, heart failure, and stroke). Atrial fibrillation and peripheral artery disease were not included, as these data were not available. |

CKD, chronic kidney disease; CKM, cardiovascular-kidney-metabolic; CVD, cardiovascular disease; NHANES, National Health and Nutrition Examination Surveys.
