# Supplementary material for: Prevalence and Correlates of Depression and Suicidal Ideation Across Stages 0–4 of Cardiovascular‐Kidney‐Metabolic Syndrome
Source: Brain Behav. 2025 Nov 11;15(11):e70989. doi: 10.1002/brb3.70989 (PMC12605968; doi:10.1002/brb3.70989)
Supplement: Supplementary file 2 — Supplementary Table: brb370989‐sup‐0002‐Table.doc [file BRB3-15-e70989-s001.doc]

Table S2. Definition of the indicators for lifestyle habits and health status

| **Variables** | **Definition** |
| --- | --- |
| ***Lifestyle habits*** | |
| Current smoking | Having smoked at least 100 cigarettes in life and now smoking cigarettes |
| Physical inactivity | Less than 150 minutes of moderate-intensity aerobic activity per week, less than 75 minutes of vigorous-intensity aerobic activity peer week, and less than an equivalent combination of moderate- and vigorous-intensity aerobic activity |
| Sleep duration | The time usually spent sleeping at night on weekdays or workdays |
| ***Health status*** | |
| Glomerular filtration rate | Calculated using the race-free CKD-EPI 2021 creatinine equation (DOI: 10.1056/NEJMoa2102953) |
| Hypertension | Having hypertension told by a doctor or other health professional, systolic blood pressure ≥ 140 mmHg, or diastolic blood pressure ≥ 90 mmHg |
| Diabetes mellitus | Having high blood cholesterol level told by a doctor or other health professional, fasting blood glucose ≥ 126 mg/dL, or hemoglobin A1c ≥ 6.5% |
| Metabolic syndrome | The presence of 3 or more of the following: (1) waist circumference ≥88 cm for women and ≥102 cm for men (≥80 cm for women and ≥90 cm for men if Asian ancestry); (2) HDL cholesterol <40 mg/dL for men and <50 mg/dL for women; (3) triglycerides ≥150 mg/dL; (4) elevated blood pressure (systolic blood pressure ≥130 mmHg or diastolic blood pressure ≥80 mmHg and/or use of antihypertensive medications); and (5) fasting blood glucose ≥100 mg/dL. |
| Cardiovascular diseases | Having congestive heart failure, coronary heart disease, angina, myocardial infarction, or stroke told by a doctor or other health professional |
| Cancer | Having cancer or a malignancy of any kind told by a doctor or other health professional |
| Arthritis | Having arthritis told by a doctor or other health professional |
| Use of antihypertensive drugs, statins, or antidepressants | Taking antihypertensive, statin, or antidepressant medications in the past month |
