# Supplementary material for: Prevalence and Correlates of Depression and Suicidal Ideation Across Stages 0–4 of Cardiovascular‐Kidney‐Metabolic Syndrome
Source: Brain Behav. 2025 Nov 11;15(11):e70989. doi: 10.1002/brb3.70989 (PMC12605968; doi:10.1002/brb3.70989)
Supplement: Supplementary file 3 — Supplementary Table: brb370989‐sup‐0003‐Table.doc [file BRB3-15-e70989-s002.doc]

Table S3. Logistic regression analysis results on the association between CKM syndrome stages and depression

| **CKM syndrome stages** | **OR** (**95% CI**) | ***p*-value*** |
| --- | --- | --- |
| Stage 0 | Ref |  |
| Stage 1 | 1.47 (1.07−2.03) | 0.019 |
| Stage 2 | 2.06 (1.53−2.77) | <0.001 |
| Stage 3 | 1.51 (0.97−2.35) | 0.068 |
| Stage 4 | 3.25 (2.26−4.67) | <0.001 |
| ***p*-value for trend** | < 0.001 |  |

*****Adjusted for age, sex, race, education level, marital status, family income-to-poverty ratio, owning house, and medical insurance coverage. CI, confidence interval; CKM, cardiovascular–kidney–metabolic; OR, odds ratio.
