# Supplementary material for: Prevalence and Correlates of Depression and Suicidal Ideation Across Stages 0–4 of Cardiovascular‐Kidney‐Metabolic Syndrome
Source: Brain Behav. 2025 Nov 11;15(11):e70989. doi: 10.1002/brb3.70989 (PMC12605968; doi:10.1002/brb3.70989)
Supplement: Supplementary file 4 — Supplementary Table: brb370989‐sup‐0004‐Table.doc [file BRB3-15-e70989-s004.doc]

Table S4. Characteristics of participants with depression stratified by CKM syndrome stages

| **Characteristic** | **Stage 0, N=97 (6.9%)** | **Stage 1, N=282 (21%)** | **Stage 2, N=892 (53%)** | **Stage 3, N=147 (5.2%)** | **Stage 4, N=308 (14%)** | ***p*-value** |
| --- | --- | --- | --- | --- | --- | --- |
| Antidepressant use (%) | 56 (71.5) | 173 (71.4) | 574 (74.9) | 105 (78.5) | 209 (77.0) | 0.57 |
| PHQ-9 score | 7.0 (1.0−11.0) | 6.0 (2.0−12.0) | 7.0 (2.0−12.0) | 6.0 (2.0−11.0) | 8.0 (3.0−13.0) | 0.26 |
| Suicidal ideation (%) | 19 (16.4) | 41 (14.5) | 137 (12.0) | 17 (13.2) | 52 (13.0) | 0.73 |

CKM, cardiovascular–kidney–metabolic; PHQ-9, nine-item Patient Health Questionnaire
